# Landau level splitting in Cd_3_As_2_ under high magnetic fields

**DOI:** 10.1038/ncomms8779

**Published:** 2015-07-13

**Authors:** Junzhi Cao, Sihang Liang, Cheng Zhang, Yanwen Liu, Junwei Huang, Zhao Jin, Zhi-Gang Chen, Zhijun Wang, Qisi Wang, Jun Zhao, Shiyan Li, Xi Dai, Jin Zou, Zhengcai Xia, Liang Li, Faxian Xiu

**Affiliations:** 1 State Key Laboratory of Surface Physics and Department of Physics, Fudan University, Shanghai 200433, China; 2 Collaborative Innovation Center of Advanced Microstructures, Fudan University, Shanghai 200433, China; 3 Wuhan National High Magnetic Field Center, Huazhong University of Science and Technology, Wuhan 430074, China; 4 Department of Materials Engineering, The University of Queensland, Brisbane, QLD 4072, Australia; 5 Beijing National Laboratory for Condensed Matter Physics and Institute of Physics, Chinese Academy of Sciences, Beijing 100190, China; 6 Centre for Microscopy and Microanalysis, The University of Queensland, Brisbane, Queensland 4072, Australia

## Abstract

Three-dimensional topological Dirac semimetals (TDSs) are a new kind of Dirac materials that exhibit linear energy dispersion in the bulk and can be viewed as three-dimensional graphene. It has been proposed that TDSs can be driven to other exotic phases like Weyl semimetals, topological insulators and topological superconductors by breaking certain symmetries. Here we report the first transport experiment on Landau level splitting in TDS Cd_3_As_2_ single crystals under high magnetic fields, suggesting the removal of spin degeneracy by breaking time reversal symmetry. The detected Berry phase develops an evident angular dependence and possesses a crossover from non-trivial to trivial state under high magnetic fields, a strong hint for a fierce competition between the orbit-coupled field strength and the field-generated mass term. Our results unveil the important role of symmetry breaking in TDSs and further demonstrate a feasible path to generate a Weyl semimetal phase by breaking time reversal symmetry.

## Abstract (web summary)

Dirac semimetals have been proposed as parent materials for other topologically non-trivial phases such as Weyl semimetals, achieved by the breaking of time reversal symmetry. Here the authors use transport measurements to evidence such behaviour in single crystal Cd_3_As_2_ under an applied magnetic field.

## Graphical abstract

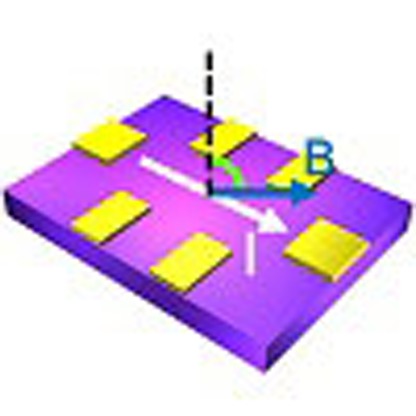

The peculiar band structure of graphene makes it a text-book Dirac material and a promising candidate for next-generation electronic devices^1^,^2^,^3^. It has been found that Dirac fermions with linear-band dispersion can give rise to various physical phenomena, such as quantum Hall effect, Andreev reflection and Klein tunnelling^1^,^2^,^4^,^5^,^6^. Driven by the excellent properties of Dirac materials, topological Dirac semimetals (TDSs), adopting a similar band structure to graphene but in the bulk form, have been theoretically proposed in several systems, including β-BiO_2_, Na_3_Bi and Cd_3_As_2_^7^,^8^,^9^. In a TDS, the conduction band and the valence band contact each other only at some discrete points (Dirac nodes) in the momentum space. These Dirac nodes are degenerated and they consist of several overlapping Weyl nodes with opposite chirality in the presence of time reversal symmetry (TRS) and inversion symmetry^7^,^8^. In the meantime, the additional crystalline point-group symmetry is required to preserve the overlapping Weyl nodes from annihilation in TDSs^8^. Therefore, the three-dimensional (3D) Dirac nodes always occur along the high-symmetry directions in the momentum space.

One of the most striking features of TDSs is the presence of various exotic phases, like Weyl semimetals, topological insulators and topological superconductors, by breaking certain symmetries in the system^8^. It has been theoretically predicted that breaking TRS or inversion symmetry can remove the degeneracy of the Dirac nodes^7^, resulting in a Weyl semimetal phase with opposite chiral Weyl node pairs^10^. Such an emerging phase promises many intriguing transport phenomena, such as chiral magnetic effect^11^ and nonlocal transport^12^,^13^, thus developing a possible basis for new electronic applications like chiral battery or quantum amplifier^11^.

Soon after the theoretical predictions, extensive experimental efforts have been devoted to the discovery of the TDS phase in the representative materials, Na_3_Bi (ref. 14) and Cd_3_As_2_ (refs 15, 16, 17, 18). Photoemission spectroscopy unveils a pair of 3D Dirac nodes in Cd_3_As_2_ locating on the opposite sides of the Brillouin zone center (Γ point) which are protected by the crystal symmetry^17^,^19^. Transport measurements reveal an ultrahigh mobility, a giant linear magnetoresistance and a nontrivial Berry phase owing to the linear-band dispersion and concomitant Dirac fermions^20^,^21^,^22^,^23^. Field-induced Landau level splitting in Cd_3_As_2_ has been observed by scanning tunnelling microscopy^15^, where a perpendicular field incurs the doublet structure of the Landau levels. However, to date, the transport experiments were mostly performed in a low magnetic field regime (<15 T) and thus unable to track the Landau level splitting in the quantum limit. In addition, a well-controlled field direction to avoid the possible crystal symmetry breaking is a prerequisite for realizing the Weyl semimetal phase, which could be accessible in the transport experiments.

In this study, we report the low-temperature magnetotransport properties of Cd_3_As_2_ single crystals under high magnetic fields. Shubnikov-de Haas (SdH) oscillations clearly resolve strong Landau level splitting at pulsed magnetic fields. The spacing of the split Landau levels, defined as the spatial difference of the split peaks, changes with the field direction, revealing a combination of the orbital and Zeeman splitting. Significantly, we observed an angular dependent Berry phase at high magnetic fields, a signature of the competition between the orbit-coupled field strength and the generated mass term. These findings serve as the evidence for the isolation of Weyl nodes and the emerging Weyl fermions in Cd_3_As_2_ by breaking the TRS.

## Results

### Structure characterizations

**Figure 1:**
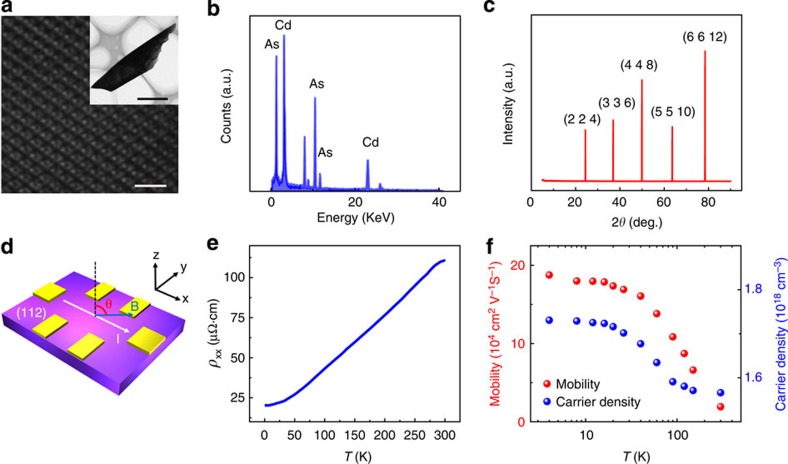
Structural and electrical properties of Cd_3_As_2_ bulk crystals. (**a**) A high-resolution TEM image of a Cd_3_As_2_ thin flake on a holey carbon grid, revealing a perfect crystalline structure. Inset is a low magnification TEM picture. The white and black scale bars correspond to 2 nm and 1 μm, respectively. (**b**) A typical EDX spectrum showing the atomic ratio of Cd:As=3:2. (**c**) X-ray diffraction patterns of the single crystal Cd_3_As_2_. The peak position shows that the sample surface is in {112} planes. (**d**) A constant current was applied within the {112} atomic planes while the magnetic field was titled in the *x*–*z* plane, as depicted by the blue arrow. (**e**) The longitudinal resistivity *ρ*_*xx*_ as a function of temperature, showing a typical metallic behaviour. (**f**) The temperature-dependent mobility and carrier density from 2.6 to 300 K. At 2.6 K, the mobility reaches 1.9 × 10^5^ cm^2^ V^−1^ s^−1^.

Transmission electron microscopy (TEM) was carried out to determine the structural characteristics of the synthesized Cd_3_As_2_ crystals. Figure 1a shows a high-resolution TEM image of a Cd_3_As_2_ thin flake on a holey carbon grid, revealing a perfect antifluorite (M_2_X)-type crystalline structure. The inset is a low magnification TEM image of the examined flake. Figure 1b shows a typical energy dispersive X-ray spectrum (EDX) with an atomic ratio of Cd:As=3:2. Consistent with the TEM results, the X-ray diffraction peaks can be indexed as series of {112} planes, which verifies the high crystallinity of the single crystals (Fig. 1c). The crystal structure of our Cd_3_As_2_ samples is found to be I4_1/acd_. Its unit cell is tetragonal with *a*=12.633(3) Å and *c*=25.427(7) Å. Each unit cell contains 96 Cd atoms and 64 As atoms.

### Hall-effect measurements

A Hall bar device with a standard six-terminal geometry was fabricated for the transport measurements, as schematically illustrated in Fig. 1d. A constant current was applied within the {112} atomic planes while the magnetic field was titled from perpendicular to parallel to the {112} planes, as depicted by the blue arrow. Figure 1e provides the temperature dependence of longitudinal resistivity *ρ*_*xx*_ at zero magnetic field. The *ρ*_*xx*_–*T* curve describes a typical metallic behaviour of Cd_3_As_2_ due to the semimetal band structure. One of the most fascinating features of Cd_3_As_2_ is the ultrahigh mobility deriving from the linear band dispersion. A typical Cd_3_As_2_ sample yields a high electron mobility of *μ*=2 × 10^4^ cm^2^ V^−1^ s^−1^ at room temperature from the Hall-effect measurements, in agreement with previous studies^22^. In fact, most of our samples (refer to Supplementary Figs 1–4 and Supplementary Table 1) have a room-temperature mobility in the range of (1∼5) × 10^4^ cm^2^ V^−1^ s^−1^. Figure 1f shows the temperature dependence of mobility (red curve), which markedly increases to 1.9 × 10^5^ cm^2^ V^−1^ s^−1^ at 2.6 K. The significant improvement of the mobility can be attributed to the alleviated phonon scattering at very low temperatures. A previous study revealed a wide-range distribution of resistivity at low temperatures in Cd_3_As_2_, corresponding to different electron mobility, which is extremely sensitive to disorder^21^. The residual resistivity of our sample (20 μΩ cm) and Hall mobility (1.9 × 10^5^ cm^2^ V^−1^ s^−1^) agree with their trend. The carrier density of the sample also exhibits a negligible change with temperature and reaches a relative low value of *n*_e_=1.67 × 10^18^ cm^−3^ at 2.6 K (Fig. 1f, blue curve). Such a low carrier density makes it easier for the Fermi level to reach low Landau levels^24^.

### Fermi surface and quantum oscillation analysis

**Figure 2:**
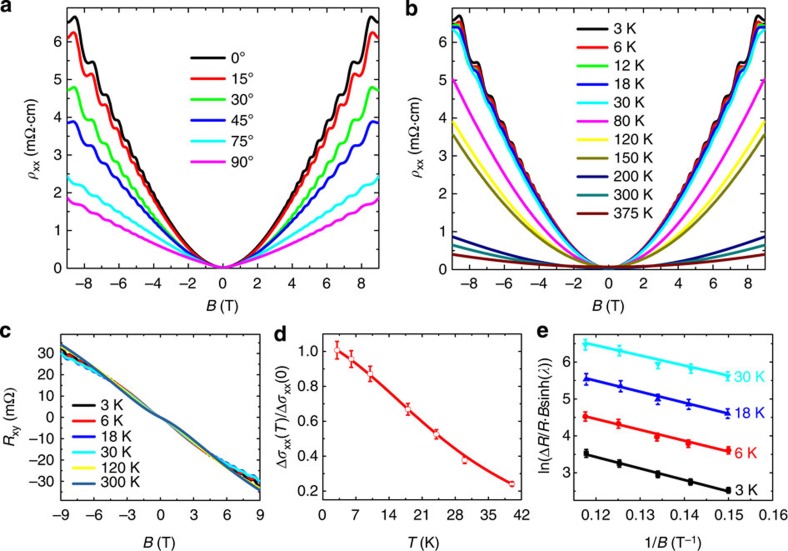
Low magnetic field transport measurements (*B*≤9 T). (**a**) The angular dependence of longitudinal resistivity *ρ*_*xx*_ at 2.6 K. The SdH oscillations are observed at different angles. The amplitude of the oscillation decreases as the angle *θ* becomes larger. (**b**) The longitudinal resistivity at different temperatures at *θ*=0°. The critical temperature is found to be 30 K, above which the oscillation is not observable. (**c**) The Hall signal *R*_*xy*_ of the sample from 3 to 300 K. (**d**) Normalized conductivity amplitude Δ*σ*_*xx*_(*T*)/Δ*σ*_*xx*_(0) versus temperature. The outcome can be fitted with the equation Δ*σ*_*xx*_(*T*)/Δ*σ*_*xx*_(0)=*λ*(*T*)/(sinh(*λ*(*T*)) and the *R*^2^ is higher than 0.999 (the coefficient of multiple determination). The error bars were estimated to be 5% of the normalized oscillation amplitude. (**e**) Dingle plots of log [Δ*R*/*R*·*B*sinh*λ*] versus 1/*B* at *θ*=0°.

To probe the Fermi surface of Cd_3_As_2_, we carried out the magnetotransport measurements using a physical properties measurement system (up to 9 T). Figure 2a,b depict the magnetoresistivity of Cd_3_As_2_ with a parabolic and a quasi-linear behaviour near the zero field and at large fields (*B*>4 T), respectively. The parabolic behaviour is originated from the orbit contribution and it becomes more pronounced at high temperatures^21^, while the quasi-linear part survives up to 375 K at large fields, although the magnetoresistivity ratio drops from 32,500% at 2.6 K to 600% at 375 K. The reduction of the magnetoresistivity ratio is believed to be associated with the temperature-sensitive phonon scattering^8^.

Apart from the giant magnetoresistivity, >90 % of our samples exhibit strong quantum oscillations, which are attributed to the Dirac band structure and the resultant ultrahigh mobility. Evident SdH oscillations can be well resolved when the temperature is below 30 K in both longitudinal magnetoresistivity and the Hall signal, as shown in Fig. 2b,c, respectively, where the oscillations can be tracked down to 3 T. The tilting angle *θ* in Fig. 2a is defined as the angle between the magnetic field *B* and the normal direction of the {112} planes (also refer to Fig. 1d). It is found that the rotation of the sample from perpendicular to parallel to the crystal plane causes the decrease of the oscillation amplitude, but the oscillation frequency remains nearly unchanged (<5%, refer to Supplementary Fig. 1b and Supplementary Note 1). The origin of the angle-dependent magnetoresistivity ratio and oscillation amplitude will be discussed later.

To fundamentally understand the SdH oscillations, we calculate the oscillation frequency (*F*) to be 61.8 T, corresponding to a periodicity of Δ(1/*B*)=0.0162 T^−1^. From the equation *F*=(*φ*_0_/2*π*^2^)*S*_F_ with *φ*_0_=*h*/2*e* (ref. 25), the cross-section area of the Fermi surface can be determined as *S*_F_=5.89 × 10^−3^ Å^−2^. Despite the strong angular dependence of the amplitude, the nearly unchanged oscillation periodicity suggests a negligible anisotropy of the Fermi sphere (Supplementary Fig. 1b). Thus, by assuming a circular cross-section, the Fermi vector of *k*_F_=0.043 Å^−1^ can be extracted. The SdH amplitude as a function of temperature can be analysed to extract key parameters of the carrier transport. The temperature-dependent amplitude Δ*σ*_*xx*_ is described by Δ*σ*_*xx*_(*T*)/Δ*σ*_*xx*_(0)=*λ*(*T*))/sinh(*λ*(*T*)), and the thermal factor is given by *λ*(*T*)=2*π*^2^*k*_B_*Tm*_cycl_/(*ħeB*), where *k*_B_ is the Boltzmann's constant, *ħ* is the reduced plank constant, and 
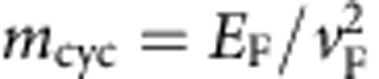
 is the cyclotron mass^26^,^27^. By taking conductivity oscillation amplitude and performing the best fit to the Δ*σ*_*xx*_(*T*)/Δ*σ*_*xx*_(0) equation, *m*_cyc_ is extracted to be 0.05 *m*_e_. Using the equation *v*_F_=*ħk*_F_/*m*_cyc_, we can obtain the Fermi velocity of *v*_F_=1.00 × 10^6^ m s^−1^ and the Fermi energy of *E*_F_=286 meV. A long mean free path of *τ*=101 nm can be estimated from the Dingle plot as shown in Fig. 2e. Table 1 summarizes the estimated parameters derived from the SdH oscillations when *B*≤9 T.

### Magnetotransport under high magnetic fields

**Figure 3:**
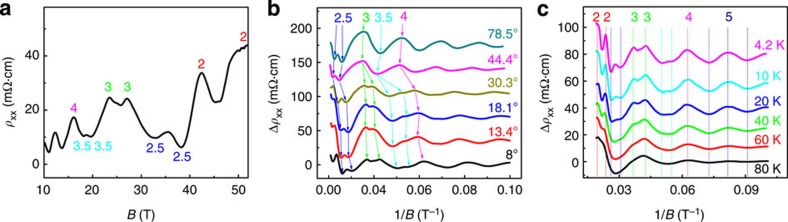
The splitting of Landau levels in the high magnetic field. (**a**) The longitudinal resistivity *ρ*_*xx*_ at *θ*=8° at 4.2 K. Landau levels are labelled by different colours with the resistivity peaks being integers and the valleys being half integers. (**b**) The angular dependence of longitudinal resistivity at 4.2 K. The SdH oscillations are observed at different angles. (**c**) The longitudinal resistivity data at different temperatures at *θ*=8°. The SdH oscillations persist up to 80 K.

To search the possible new phases involving symmetry breaking, it is necessary to apply higher magnetic field to reach the lower Landau levels. Indeed, a pulsed field of 52 T drives the sample to the second Landau level, as shown in Fig. 3a, where the longitudinal resistivity is plotted against the magnetic field (up to 52 T). The angular and temperature dependence of high field longitudinal resistivity along with the related Landau fan diagrams are plotted in Supplementary Figs 5–7. Here we use integers to denote peaks and half integers to represent valleys^25^, from which the splitting of the second and the third Landau levels can be clearly witnessed. The Landau level splitting observed here is considered to be the joint effect of the Zeeman and the orbit contributions^15^. It has been theoretically predicted that under a relatively large magnetic field, the Fermi surface topology as well as the topological charge enclosed by the Fermi surface can be largely tuned by varying the field strength and direction^9^. Thus the TDS system shows a variety of distinct topological phase transitions driven by breaking symmetries^9^,^28^. When a magnetic field is applied, the TRS in the system is no longer preserved^9^,^24^. By considering the exchange couplings induced by the external field, we can in general separate the field-dependent Hamiltonian to the orbital-dependent part and the orbital-independent part as *H*_ex1_=*h*_1_*σ*_*z*_⊗*τ*_z_ and *H*_ex2_=*h*_2_*σ*_*z*_⊗*I*, respectively^9^,^15^, where *h*_1_ and *h*_2_ are the field strength along the *z* direction, *σ*_*z*_ and *τ*_*z*_ are Pauli matrices for spin and pseudospin, respectively. If the field only couples to spin (*H*_ex1_=0), the Fermi surface will split into two concentric spheres. If the field couples to spin and orbit both (*H*_ex1_≠0), the Fermi surface will split into two separate Weyl pockets^9^,^21^. Furthermore, if the perturbation of the field on the crystal symmetry is considered, a mass term accompanied by the gap opening will be introduced to the Weyl nodes. Thus there will be a competition between the orbit-coupled field strength *h*_1_ and the field-generated mass term *m*. When *h*_1_ is larger than *m*, the Weyl semimetal phase can be developed^9^.

To clarify the respective contributions of the orbit and the Zeeman term to the Landau level splitting, we performed the angle-dependent magnetotransport measurements under high fields. In Fig. 3b, when tilting the sample from *θ*=8° to 78.5°, the splitting features remain well resolved and the split spacing changes with *θ*. Theoretically, it was predicted that the orbital-dependent splitting is highly sensitive to the field direction, while the Zeeman term shows no significant angular dependence when the field strength is fixed^15^. Also, the orbital-dependent splitting can reach the maximum when the field is along the [001] direction and vanishes when the field is perpendicular to the [001] direction^15^. In our experiments (Fig. 3b), the split spacing for the 2.5th peak shows less angular dependence than that of the 3rd and the 3.5th peaks. Thus, the Zeeman term presumably dominates in the 2.5th peak splitting, while for the 3rd and the 3.5th peaks the orbital term has a large contribution. The Landau level splitting in another sample also shows a strong angular dependence as shown in Supplementary Fig. 8.

Figure 3c displays the longitudinal magnetoresistivity oscillations under high magnetic fields at different temperatures (refer to Supplementary Fig. 7 for the original data). The splitting of the Landau levels becomes less resolved with increasing temperature. Owing to the large effective Landé factor 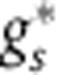 in Cd_3_As_2_ 
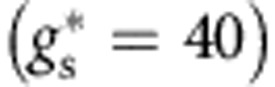
15,22, the obtained Zeeman splitting energy 
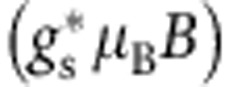
 should be considerably large, especially in the high magnetic fields (corresponding to the lower bands)^24^. When raising the temperature, the thermal energy *k*_B_*T* of electrons becomes larger, and in some materials it turns to be comparable to the Zeeman splitting energy. Between 4.2 and 80 K, the splitting of the lowest two levels remains evident primarily due to the fact that the splitting energy (from Zeeman and orbit) at high fields is still larger than the thermal energy *k*_B_*T*.

**Figure 4:**
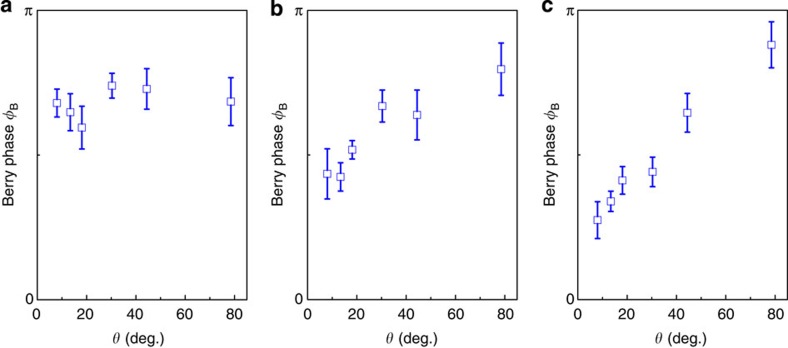
The angular dependent Berry phase Φ_B_ at different magnetic field regimes. The Berry phase was fitted in the regimes of (**a**) 5–7 T (corresponding to nineth to eleventh Landau levels); (**b**) 7–10 T (seventh to nineth Landau levels); and (**c**) 10–15 T (fifth to seventh Landau levels). The Berry phase develops an angular dependence at high magnetic fields, suggesting a field-induced phase transition. The error bars were generated from the linear fitting process in the Landau fan diagrams.

In a Dirac system, there exists a ‘zero mode' that does not shift with the field, leading to a nontrivial *π* Berry phase^24^,^29^,^30^. According to the Lifshitz–Onsager quantization rule: 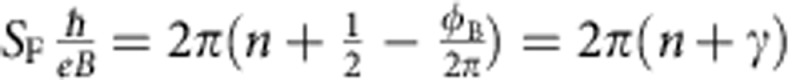
29, the offset *γ* in the Landau fan diagram gives the Berry phase *φ*_B_ by 
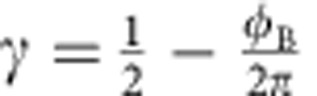
, where *ħ* is the reduced Planck's constant and *e* is the elementary charge. For a nontrivial Berry phase, *γ* should be 0 or 1. However, the exchange couplings with the magnetic field (including the Zeeman and the orbital terms) lift the degeneracy at the Dirac point. As aforementioned, the field will affect the crystal symmetry if the direction is not along the [001] direction. In this scenario, a gap will emerge as long as the orbit-coupled field strength is smaller than the mass term, resulting in the shift of the Berry phase. Consequently, a change in the Berry phase can be expected at higher fields^24^. Here we extrapolate the value of the offset *γ* in the field regimes of 5–7 T (corresponding to nineth to eleventh Landau levels), 7–10 T (seventh to nineth Landau levels) and 10–15 T (fifth to seventh Landau levels). The reason we choose the regime of *B*≤15 T to perform the Berry phase fitting is that at high fields the Landau fan diagram itself becomes nonlinear and inevitably introduces a large deviation in the linear fitting process (see Supplementary Fig. 6 and Supplementary Note 2 for more details). From the offset, the Berry phase *φ*_B_ can be acquired via the Lifshitz–Onsager equation, as summarized in Fig. 4, where *φ*_B_ gradually develops an angular dependence at high field regimes. In comparison, the corresponding Berry phase shows no such dependence at the low field regime (5–7 T). Taking *θ*=8° as an example, the Berry phase changes from (0.67±0.05)*π* to (0.27±0.06)*π* when the external field increases. Apparently, our experiments show a tendency that the Berry phase changes from nontrivial to trivial as the magnetic field is increased when *θ* is small.

## Discussion

It has been demonstrated that an additional phase shift will arise from the curvature of the Fermi surface, changing from 0 for a quasi-2D cylindrical Fermi surface to 
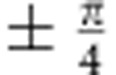
 for a corrugated 3D Fermi surface, with the precise value determined by the degree of two-dimensionality^25^. However, from our results, the disparity of the Berry phase is much larger than 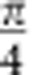. And the Berry phase acquired from the low-field regime does not have such a crossover from nontrivial phase to trivial state at different field directions. It suggests that the angular dependent Berry phase is related to a field-generated phase transition instead of the Fermi surface curvature. The Berry phase tends to be trivial at small tilting angles, while it retains nontrivial at large angles (for example, *θ*=78.5°). No aperiodic behaviour was observed at *θ*=78.5° in our field range (refer to Supplementary Fig. 6). These evidences suggest that when *θ* is 78.5°, the system remains nontrivial states and no gap is induced even under high magnetic fields. It should be noted that the field is not applied along the [001] direction when *θ*=78.5°, in which the crystal symmetry is expected to be broken and a mass term could be in principle introduced to the system. Although we cannot fully exclude other possible mass-generation mechanisms in the system, which could be induced by high magnetic fields, this new dependence of Berry phase on field direction and strength matches the phase diagram proposed in previous theoretical study^9^. Therefore, the presence of the nontrivial phase at *θ*=78.5° in our experiments provide evidence that the field-generated mass term could be removed through the possible formation of a Weyl semimetal phase, consistent with the recent predictions^7^,^9^,^31^.

After clarifying the field effect on the Cd_3_As_2_ crystal, we revisit the observed linear magnetoresistivity. According to the recent study, the giant linear magnetoresistivity in TDSs may result from a new mechanism against backscattering collapsing in the presence of the magnetic field^21^. As discussed previously, the magnetic field can break the TRS and affect the crystal symmetry simultaneously^9^. The crystal symmetry breaking will generate a gap in the Dirac nodes^9^,^15^. By controlling the field direction, we can induce discrete Weyl nodes to retain the gapless feature and nontrivial Berry phase. However, as long as the orbital-dependent splitting is strong enough to eliminate the induced gap, a Weyl semimetal phase can still emerge and the protection from backscattering survives^9^. Owing to the field-direction-sensitive nature of the orbital-dependent splitting, the magnetoresistivity ratio and the oscillation amplitude are closely related to the field direction as well.

In conclusion, we observe the Landau level splitting and an altered Berry phase under the high magnetic fields in the ultrahigh mobility Cd_3_As_2_ single crystals. The orbital-dependent splitting and the Berry phase can be significantly affected by the direction of the applied field. Our study demonstrates the possibility of inducing Weyl semimetal phase in TDSs by breaking symmetries. Further improvement could be accomplished by using local magnetic dopants to achieve an intrinsic Weyl semimetal. After submitting the manuscript, we became aware of the related studies reporting the observation of an intrinsic Weyl semimetal in TaAs class^32–35^.

## Methods

### Single-crystal growth

High-quality Cd_3_As_2_ single crystals were synthesized by self-flux growth method in a tube furnace. Stoichiometric amounts of high-purity Cd powder (4 N) and As powder (5 N) elements were placed inside an alumina crucible. The molar ratio of Cd and As was 8:3. After mixing two elements uniformly, the alumina crucible was sealed in an iron crucible under argon atmosphere. The iron crucible was heated to 800–900 °C and kept for 24 h, then slowly cooled down to 450 °C at 6 °C h^−1^. Next, the crucible was kept at 450 °C for >1 day then cooled naturally to room temperature. The superfluous Cd flux was removed by centrifuging in a vacuum quartz tube at 450 °C.

## Additional information

**How to cite this article:** Cao, J. *et al.* Landau level splitting in Cd_3_As_2_ under high magnetic fields. *Nat. Commun.* 6:7779 doi: 10.1038/ncomms8779 (2015).

## Supplementary Material

Supplementary Information Supplementary Figures 1-8, Supplementary Table 1, Supplementary Notes 1-2 and Supplementary References.

## Figures and Tables

**Table 1:** Estimated parameters from the SdH oscillations (*B*≤9 T).

	m^*^	***S***_**F**_ **(Å**^**−2**^**)**	***k***_**F**_ **(Å**^**−1**^**)**	***t*** **(s)**	***v***_**F**_ **(m s**^**−1**^**)**	***l*** **(nm)**	***E***_**F**_ **(meV)**
**Sample 1**	0.05*m*_0_	5.89 × 10^−3^	0.043	1.41 × 10^−13^	1.00 × 10^6^	101	286

Transport parameters including the effective mass m*, Fermi surface *S*_F_, Fermi vector *K*_F_, carrier lifetime *t*, Fermi velocity *v*_F_, mean free path *l*, and Fermi energy *E*_F_, can be extracted from the SdH oscillations.
