# Supplementary material for: Landau level splitting in Cd3As2 under high magnetic fields
Source: Nat Commun. 2015 Jul 13;6:7779. doi: 10.1038/ncomms8779 (PMC4510959; doi:10.1038/ncomms8779)
Supplement: Supplementary Information — Supplementary Figures 1-8, Supplementary Table 1, Supplementary Notes 1-2 and Supplementary References. [file ncomms8779-s1.pdf]

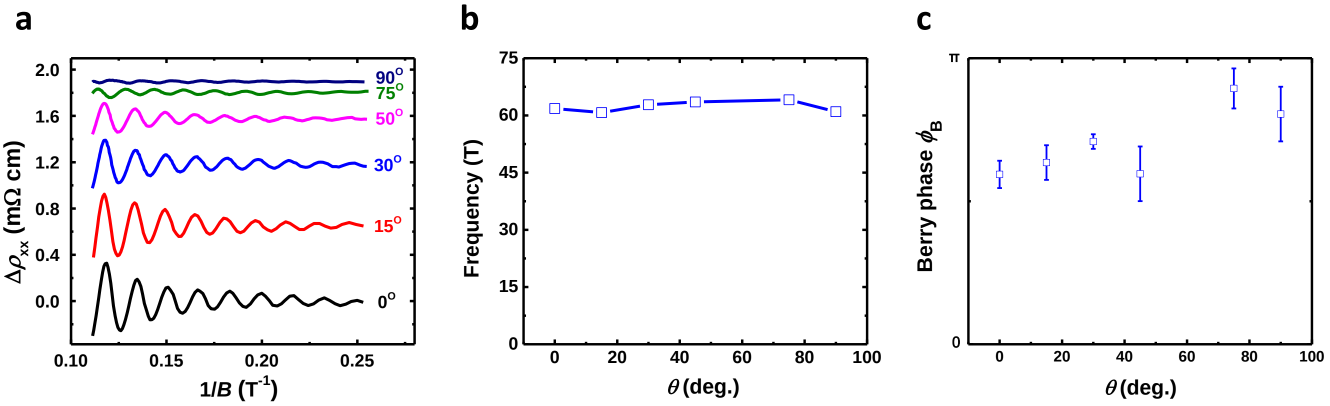

Supplementary Figure 1. Amplitude and frequency of oscillations and the fitted Berry phase at different angles. a, Amplitude of Shubnikov-de Haas (SdH) oscillations $\Delta\rho_{xx}$ at different angles. Background signal is removed. The curves are vertically shifted for clarity. **b,** Oscillation frequency at different angles. The anisotropy of frequency is negligible (within 5%). **c**, The fitted Berry phase Φ_B from the low field regime (B<9 T) at different field directions. Note that this is the Sample 1 from the main text. The error bars were generated from the linear fitting process in the Landau-fan diagrams.

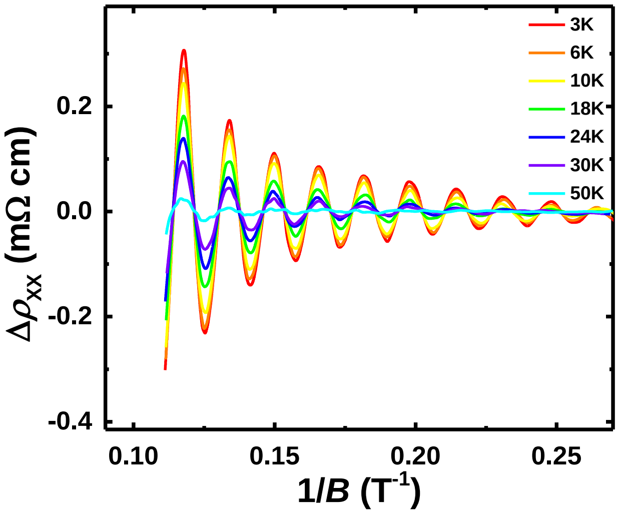

Supplementary Figure 2. Amplitude of oscillations at different temperatures. Oscillation amplitude of longitudinal resistivity at different temperatures with a single frequency of 61.8 T. Note that this is the Sample 1 from the main text.

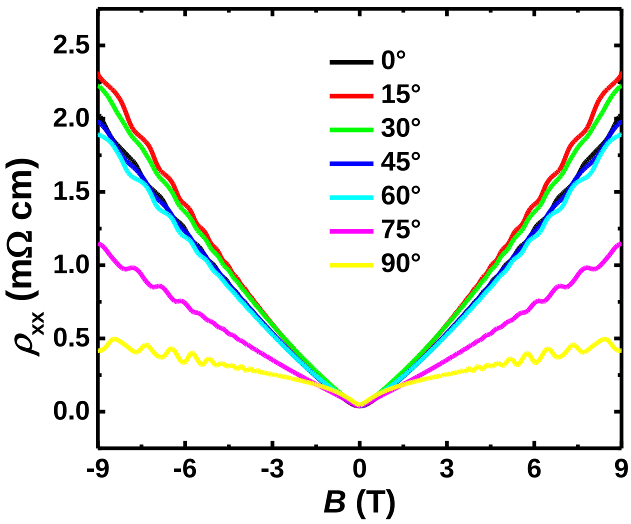

Supplementary Figure 3. Low field longitudinal resistivity ρ_{xx} of Sample 2. The Hall mobility of this sample is 2.5×10^{5} cm^{2} V^{-1} s^{-1} at 2.6 K. Room-temperature mobility is 4.0×10^{4} cm^{2} V^{-1} s^{-1}.

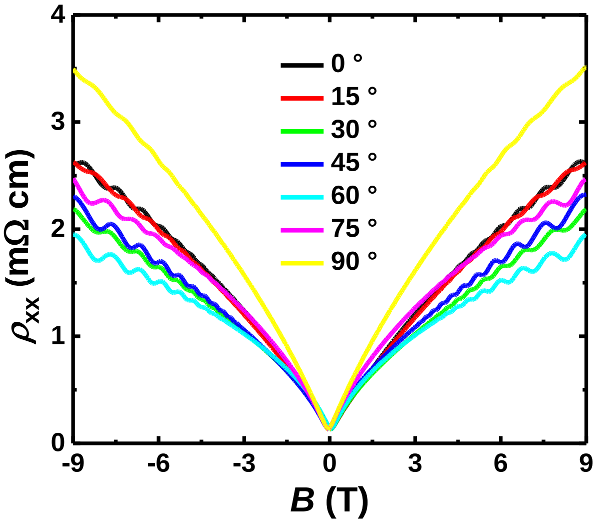

Supplementary Figure 4. Low field longitudinal resistivity ρ_{xx} of Sample 3. The Hall mobility of this sample is 1.6×10^5 cm^2 V^{-1} s^{-1} at 2.6 K. Room-temperature mobility is 4.8×10^4 cm^2 V^{-1} s^{-1}.

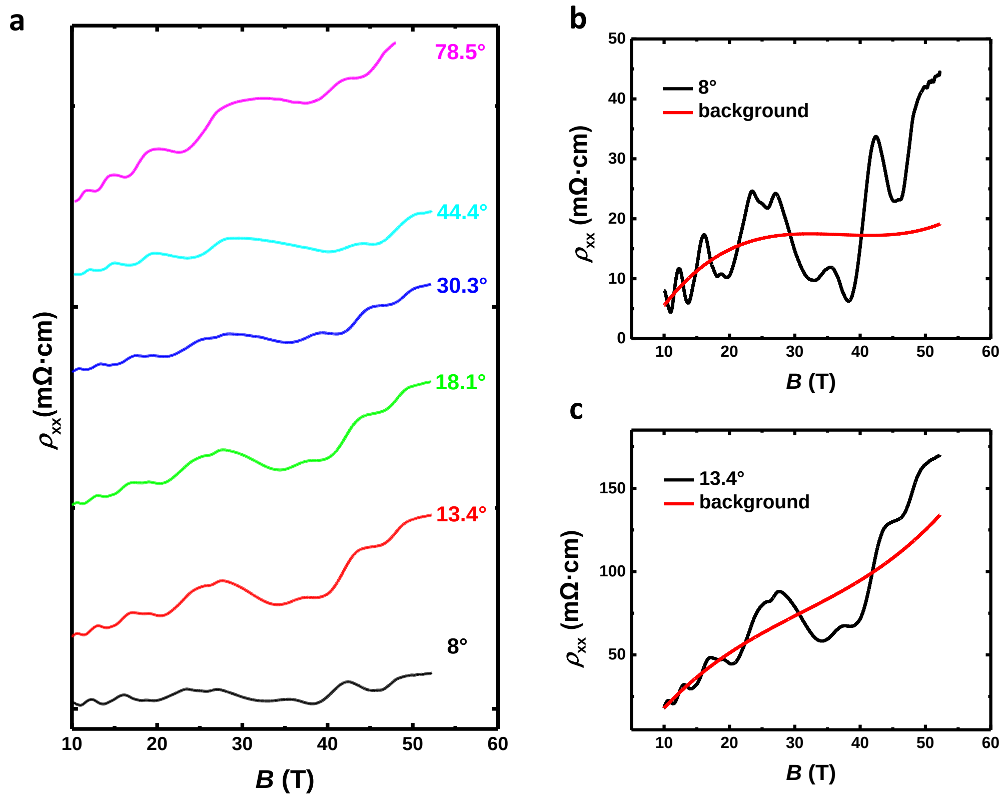

Supplementary Figure 5. High field longitudinal resistivity ρ_{xx} of Sample 1. a, The splitting phenomenon observed at different angles. **b** and **c**, The raw magnetoresistivity data (black line) and the fitted magnetoresistivity background (red line) for θ=8° and 13.4^{o}, respectively.

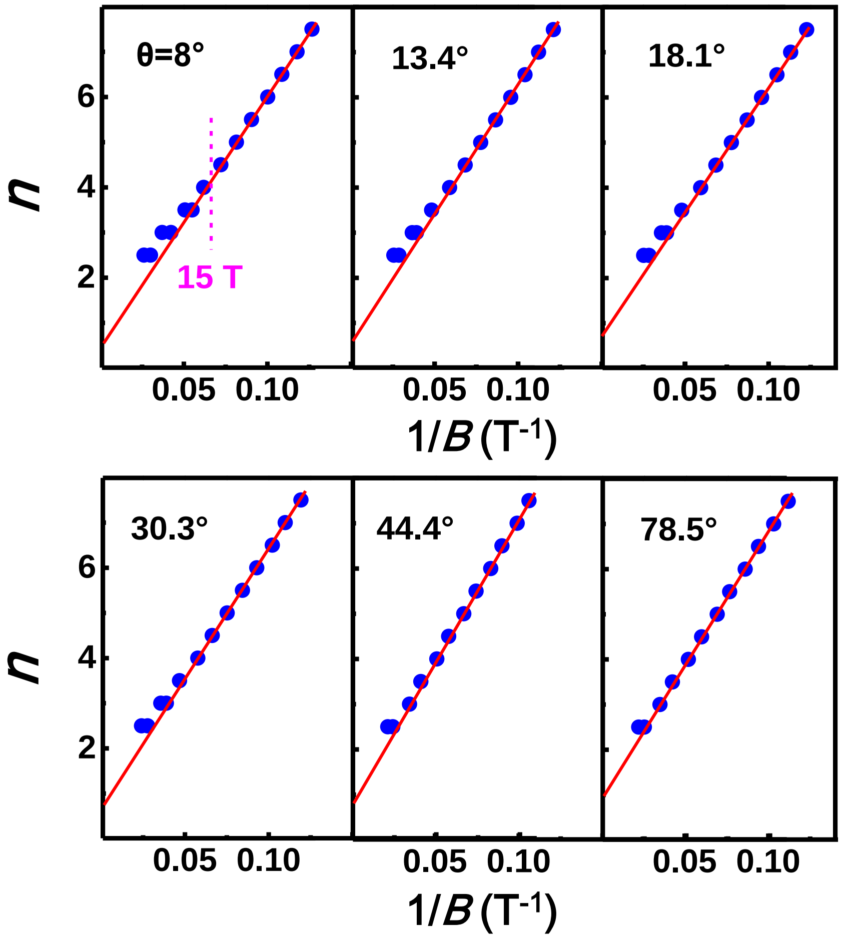

Supplementary Figure 6. The deviation of the linear relation in Landau fan diagrams. Landau level index *n* with respect to 1/*B*. At relative small fields ($B \leqslant 15$ T), the linear relation is evident. However, at relative large magnetic fields ($B > 15$ T), the Landau Level splitting occurs and the peak position moves to the same side of the red lines, which indicates the alternation of the Berry phase.

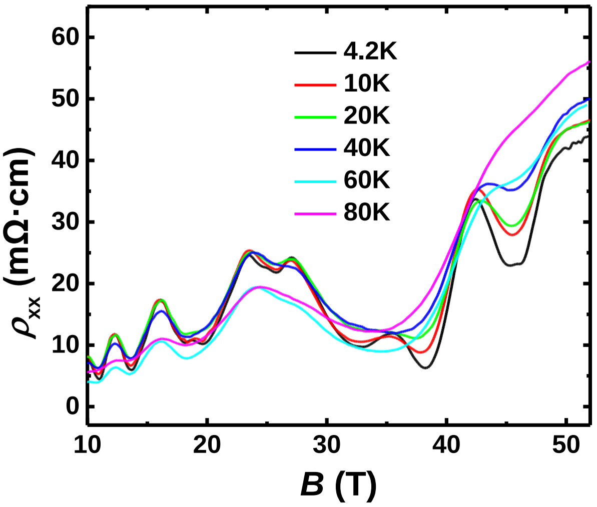

Supplementary Figure 7. High-field longitudinal resistivity at different temperatures. The splitting of Landau Level at different temperatures at a fixed tilting angle of 8°. Positions of all peaks and valleys remain the same at different temperatures. It is clearly shown that the splitting for the relative higher Landau levels smear out as the temperature increases.

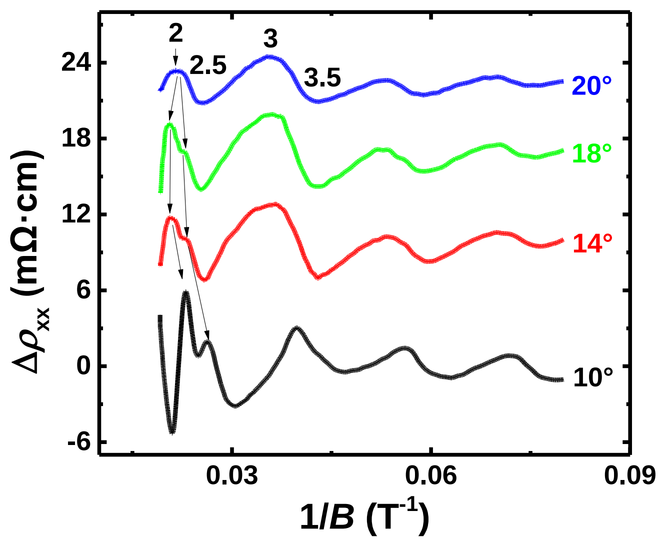

Supplementary Figure 8. Splitting of the 2nd Landau level of Sample 2 under high magnetic fields. The curves are vertically shifted for clarity.

	m^*	S_F (Å^{-2})	K_F (Å^{-1})	t (s)	v_F (m/s)	l (nm)	E_F (meV)
**Sample 2**	0.043 m_0	5.55×10^{-3}	0.042	1.07×10^{-13}	1.12×10^{6}	119	309
**Sample 3**	0.050 m_0	5.51×10^{-3}	0.042	4.94×10^{-14}	9.68×10^{5}	47.8	268

Supplementary Table 1. Estimated parameters of Sample 2 & Sample 3. Transport parameters including the effective mass m^*, Fermi surface S_F, Fermi vector K_F, carrier lifetime t, Fermi velocity v_F, mean free path l, and Fermi energy E_F, can be extracted from the SdH oscillations.

Supplementary Note 1. Low magnetic field transport (B≤9 T)

To understand the properties of bulk electrons and the structure of Fermi sphere, we analyze the SdH oscillations under low fields (B≤9 T) in a physical properties measurement system (PPMS). The longitudinal resistivity is shown in the main text (Figure 1e). In order to accurately identify the position of the oscillation peaks and valleys, we remove the background signal (Supplementary Figure 1a), from which the oscillation frequency F at different angles can be obtained via the equation $F = \frac{\phi_0}{2\pi^2} S_{\mathrm{F}}$, where $\phi_0 = h/2e$ and S_{F} is the cross-section area of the Fermi surface perpendicular to the magnetic field. Thus the frequency of oscillations at different angles can be used to estimate the configuration of Fermi sphere. Supplementary Figure 1b displays the oscillation frequency at different angles. The anisotropy is within 5%. This negligible anisotropy points to a nearly uniform Fermi sphere of Cd_3As_2, which is consistent with the recent photoemission results.[1]

The low-temperature mobility of our sample matches the range of the reported values (10^4~10^7 cm^2 V^{-1} s^{-1}).[2, 3, 4, 5, 6] We noticed that the Hall mobility of Cd_3As_2 bulk materials presented in most studies falls in the range of (10^4~10^5 cm^2 V^{-1} s^{-1}). The reason for the large distribution is that the mobility is extremely sensitive to disorders.[4] A previously systematic investigation revealed the relationship between the low temperature residual resistivity and mobility. They found that the lower the residual resistivity was, the higher the mobility would be. The residual resistivity of our sample (20 μΩ cm) and Hall mobility (1.9×10^5 cm^2 V^{-1} s^{-1}) agrees with the trend.[2]

Supplementary Note 2. High magnetic field transport and the SdH oscillation analysis

In the main text, Figure 3b and 3c show the SdH oscillation amplitude under high magnetic fields. The original data are displayed in Supplementary Figure 5 and 7. Supplementary Figure 5 shows the longitudinal resistivity at different angles. As the direction of magnetic field tilts towards in-plane direction, the splitting becomes less evident. In Supplementary Figure 7, the longitudinal resistivity at different temperatures is plotted on the same graph. As the temperature increases, the splitting of higher Landau levels becomes less evident. However, the splitting of the 2nd Landau level remains well-resolved. The reason is that the splitting energy at this high magnetic field is large enough to overcome the thermal energy.

Supplementary Figure 6 summarizes the Landau-fan diagrams at different tilting angles. It shows that the non-split Landau levels (higher than the 4th Landau level) are exactly on the same positions regardless of θ, suggesting that the Fermi surface barely changes below 15 T. However, beyond 15 T the Landau fan diagram develops angle-dependent anisotropy, which can be observed from the deviation of the points to the linear-fit line in Supplementary Figure 6 (Red lines are the fitted background curves). The Landau level index n shows clear aperiodic feature when approaching the split Landau levels at small θ. As the direction of the magnetic field tilts towards 90°, however, the deviation becomes less evident: the positions of the

peaks and the valleys at large fields almost completely collapse to the line when θ equals to 78.5^{o}.

The reason we choose the regime of $B \leq 15$ T to perform the Berry phase fitting is that at high fields the Landau fan diagram itself becomes nonlinear and inevitably introduces a large deviation in the linear fitting process. To be explicit on this point, Supplementary Figure 6 reveals such effect that the Landau levels at higher magnetic fields ($B>15$ T) adopt a nonlinear trend especially at small angles. In addition, the splitting of Landau levels at high fields makes it even harder to trace the original peak position. Therefore, it is certainly not rational to fit the Landau fan diagram with the magnetic field over 15 T in our scenario. We note that the same practice has been exercised in the previous study of topological insulators where they used a specific fitting range of 2nd to 4th Landau level to derive the altered Berry phase at high fields.[7]

In other samples, the splitting of Landau level is also observed under high magnetic fields as shown in Supplementary Figure 8. This is the same sample as used in Supplementary Figure 3 (Sample 2). We can clearly see the splitting of the 2nd Landau level. Splitting of higher Landau levels is less evident. It is possibly because of the surface orientation of this sample. Also, Landau level splitting is not observed in the samples with high Fermi energy (high carrier concentrations).

Supplementary References

1. Liu, Z. K., *et al.* A stable three-dimensional topological Dirac semimetal Cd_3As_2. *Nat. Mater.* **13**, 677-681 (2014).

2. Liang, T., Gibson, Q., Ali, M. N., Liu, M., Cava, R. J., Ong, N. P. Ultrahigh mobility and giant magnetoresistance in the Dirac semimetal Cd_3As_2. *Nat. Mater.* **14**, 280-284 (2014).

3. He, L. P., *et al.* Quantum Transport Evidence for the Three-Dimensional Dirac Semimetal Phase in Cd_3As_2. *Phys. Rev. Lett.* **113**, 246402 (2014).

4. Narayanan, A., *et al.* Linear Magnetoresistance Caused by Mobility Fluctuations in n-Doped Cd_3As_2. *Phys. Rev. Lett.* **114**, 117201 (2015).

5. Spitzer, D. P. Anomalous Thermal Conductivity of Cd_3As_2 and the Cd_3As_2–Zn_3As_2 Alloys. *J. Appl. Phys.* **37**, 3795 (1966).

6. Feng, J., *et al.* Large linear magnetoresistance in Dirac semi-metal Cd_3As_2 with Fermi surfaces close to the Dirac points. *Preprint at http://arxiv.org/abs/1405.6611*, (2014).

7. Wang, Z., *et al.* Dirac semimetal and topological phase transitions in A_3Bi (A=Na, K, Rb). *Phys. Rev. B* **85**, 195320 (2012).
